# A Coded Aperture Compressive Imaging Array and Its Visual Detection and Tracking Algorithms for Surveillance Systems

**DOI:** 10.3390/s121114397

**Published:** 2012-10-29

**Authors:** Jing Chen, Yongtian Wang, Hanxiao Wu

**Affiliations:** Key Laboratory of Photoelectronic Imaging Technology and System, Ministry of Education of China, School of Optoelectronics, Beijing Institute of Technology, Beijing 100081, China; E-Mails: wyt@bit.edu.cn (Y.W.); whx0647@163.com (H.W.)

**Keywords:** compressive imaging, coded aperture, compressive sensing, motion detection and tracking

## Abstract

In this paper, we propose an application of a compressive imaging system to the problem of wide-area video surveillance systems. A parallel coded aperture compressive imaging system is proposed to reduce the needed high resolution coded mask requirements and facilitate the storage of the projection matrix. Random Gaussian, Toeplitz and binary phase coded masks are utilized to obtain the compressive sensing images. The corresponding motion targets detection and tracking algorithms directly using the compressive sampling images are developed. A mixture of Gaussian distribution is applied in the compressive image space to model the background image and for foreground detection. For each motion target in the compressive sampling domain, a compressive feature dictionary spanned by target templates and noises templates is sparsely represented. An *l*_1_ optimization algorithm is used to solve the sparse coefficient of templates. Experimental results demonstrate that low dimensional compressed imaging representation is sufficient to determine spatial motion targets. Compared with the random Gaussian and Toeplitz phase mask, motion detection algorithms using a random binary phase mask can yield better detection results. However using random Gaussian and Toeplitz phase mask can achieve high resolution reconstructed image. Our tracking algorithm can achieve a real time speed that is up to 10 times faster than that of the *l*_1_ tracker without any optimization.

## 1. Introduction

In the field of computer vision, video surveillance is always an important tool in a variety of security applications. The challenge in video surveillance systems is that the use of conventional imaging approaches in such applications can result in overwhelming data bandwidths. To solve this problem, researchers generally compress those high-resolution video streams by using various data compression algorithms to reduce the overall bandwidth to a more manageable level. However, the optics and photo detector hardware must still operate at the native bandwidth, which seriously wastes valuable sensing resources and increases overall system cost. In fact, in video surveillance systems moving objects occupy only a small part of the full image, and a large portion of any obtained image data is redundant, such as the static background in the field of view that is repeated in every frame. We thus pose the following question: could we directly obtain compressed images during the collection process while ensuring that relevant information is preserved, only using these compressive measurements for detection and tracking of objects in motion?

The new emerging theory of compressive sensing (CS) demonstrates that it is possible to reconstruct signals perfectly or robustly approximated with far fewer samples than the Shannon sampling theorem implies, when signals are sparse in some linear transform domain [1,2]. In fact, almost all images are sparse and compressible. Based on this assertion, a new research direction on compressive imaging (CI) has been developed [3]. The objective of a compressive imager is to design optical sensors that can collect linear random projections of a scene onto a small focal plane array and allow sophisticated computational methods to be used to recover the original scene image. CI has valuable implications for image acquisition fields, especially in fields with limited power, communication bandwidth and image sensor hardware, such as distributed camera networks, camera arrays and IR or UV cameras, and several promising compressive optical imaging architectures have been proposed. Although the field of CI is rapidly becoming viable for real-world sensing applications, little attention has been paid on motion target detection and tracking by using compressive sampling images, which could be an important application field of practical compressive imaging systems. In this paper, our goal is to optimize the optical CS imaging process not only to collect data in a compressed format, but also to perform motion target detection and tracking algorithms directly in a CI surveillance system.

The main contributions of this research can be summarized in the following three aspects: first, we propose a coded aperture lens array optical system to realize CS imaging. This architecture can effectively reduce the needed high-resolution coded mask requirements and facilitate the storage of the projection matrix. Second, we describe a motion detection algorithm that is directly employed by using CI data without recovering traditional images. A mixture of Gaussian distribution is applied to model the background image directly in the CS space. Third, a real-time CS *l*_1_ tracking algorithm which is 10 times faster than the *l*_1_ tracking method is proposed.

The rest of this paper is organized as follows: in Section 2 the related work on the compressive sensing theory, state of the art CS imaging and motion detection and tracking algorithms using CS theory is reviewed. In Section 3, CS imaging based on the coded aperture lens array system is discussed. In Sections 4 and 5, motion detection and tracking algorithms applied directly on compressive sampling space are exploited. Experimental results for our CI optical system and the motion detection and tracking methods are presented in Section 6. In Section 7 we draw some conclusions from the results of our simulation study.

## 2. Related Work

### 2.1. Background of CS

Consider a scene represented as a vector *X* of length *N*. The CI camera observes the scene and generates a measurement vector *Y* of length *M*. In a noise free scenario, each of the *M* elements in the measurement *Y* represents a projection of the scene *X* onto the basis vectors comprising the projection matrix Φ. In matrix vector form, this set of linear equations can be expressed as:

(1) $$\left[\begin{array}{c} y_{1}\\ y_{2}\\ \vdots \\ y_{m} \end{array}\right]=\left[\begin{array}{ccccc} \Phi_{11} & \Phi_{12} & \ldots & \ldots & \Phi_{1n}\\ \Phi_{21} & \Phi_{22} & \ldots & \ldots & \Phi_{2n}\\ \vdots & \vdots & \ddots & & \vdots \\ \Phi_{m1} & \Phi_{m2} & & & \Phi_{mn} \end{array}\right]\left[\begin{array}{c} x_{1}\\ x_{2}\\ \begin{array}{l} \vdots \\ \vdots \end{array}\\ x_{n} \end{array}\right]$$

or:

(2) Y=ΦX

where the dimensions of the projection matrix Φ are *M* × *N*, and each row of Φ represents a sampling of the underlying image signal. If image signals are sparse, such signals can be expressed by a set of coefficients θº*R^N^* in some orthonormal basis ψ ∈ *R^N^*^×^*^N^*:

(3) X=Ψθ

In many cases, the basis ψ = [*ψ*_1_*ψ*_2_ … *ψ_n_*] can be chosen so that only *K* ≪ *N* coefficients have significant magnitude. The image signal can be called K-sparse. The key principle of CS is that, with slightly more than *K* well-chosen measurements, a K-sparse signal can be recovered by multiplying it by a random projection matrix Φ*_M_*_×_*_N_*. Here *M* is significantly smaller than *N* but larger than *K*. Substituting Equation (3) into Equation (2) we observe that:

(4) Y=ΦX=ΦΨθ

CS addresses the problem of solving for *X* when the measurements are much smaller than original image signals. This is generally an ill-posed problem, because there are an infinite number of candidate solutions for *X*. Nevertheless, the CS theory provides a set of conditions that, if *X* is sparse or compressible in a basis ψ, and Φ in conjunction with ψ satisfies a technical condition called the Restricted Isometry Property (RIP):

(5) $$\left(1-\delta \right){\left\|x\right\|}_{2}^{2}\leq {\left\|\Phi \Psi x\right\|}_{2}^{2}\leq \left(1+\delta \right){\left\|x\right\|}_{2}^{2}$$

Candes and Tao [4,5] show that the signal *X* can be exactly recovered from few measurements by solving a *l*_2_ – *l*_1_ minimization problem:

(6) $$\hat{x}=\arg\min\frac{1}{2}{\left\|y-\Phi x\right\|}_{2}^{2}+\lambda {\left\|\Psi^{T}x\right\|}_{1}$$

Here the regularization parameter λ > 0 helps to overcome the ill-posed problem, and the *l*_1_ penalty term drives small components of *θ* to zero and helps promote sparse solutions. In fact, the RIP constrained condition of Equation (5) suggests that the energy contained in the projected image *Y* is close to the energy contained in the original image *X*.

### 2.2. CI

Compared with conventional camera architectures, the CI camera is specifically designed to exploit the CS framework for imaging. For example, the single pixel camera designed by Rice University differs fundamentally from a conventional camera [6]. A programmed digital micro-mirror device is used to perform linear projections of an image onto a single optical photodiode. In this type of optical architecture, the system cycles sequentially through the rows of the projection matrix Φ to determine the measurement elements one at a time. Any arbitrary pattern of values in the domain [0,1] can be easily used by reprogramming the control software. However, as the measurement elements of *y* are measured sequentially, dynamic imaging is inherently time consuming. Considering the dynamic scene imaging problem, researchers have proposed some other optical CI systems. Rather than measuring a sequence of a scene image to a single pixel, they make a parallel measurement of the original scene image onto a small set of pixels. For example, the Duke University group describes the design of coded aperture masks for super resolution image reconstruction from a single, low-resolution, noisy observation image [7,8]. This architecture is simple and highly suitable for optical CS imaging because all measurements are collected at one time. More recently, based on their prior work, Harmany *et al.*[9] proposed a coded aperture keyed exposure sensing paradigm to realize spatio-temporal compressive sensing imaging. However, how to make the random coded aperture practically remains a key problem that needs to be solved. Fergus *et al*. reported a compact CI camera that uses a random lens [10]. This approach can achieve an ultra-thin optical system design and can be applied to numerous practical applications. However obtaining the sensing matrix from these random lenses is difficult. Shi *et al.*[11] proposed a compressive optical imaging system based on spherical aberration. Spherical aberration is an optical phenomenon attributed to the intrinsic refraction property of a spherical lens. The larger the curvature of the lens surface, the more serious the aberration will be. The optical structure of this architecture only needs a lens with significant spherical aberration. Although the research on this method is being undertaken, the method by which to design and to manufacture this special lens may be not easy. In [12,13], Neifeld *et al*. proposed an adaptive feature-specific imaging system for face recognition tasks.

In summary, all the aforementioned compressive sampling strategies satisfy the following features: each element *x_i_* in the source image contributes to all compressed measurements {*y*_1_*y*_2_ … *y_m_*} and each compressed measurement *y_i_* is a linear combination of all source elements {*x*_1_*x*_2_ … *x_n_*}. The coding of a particular pixel *y_i_* is relatively uncorrelated with that of its neighbors.

### 2.3. Motion Targets Detection and Tracking by Using CS

In surveillance systems, background subtraction is commonly used for segmenting out objects of interest in a scene. However background subtraction techniques may require complicated density estimates for each pixel, which become burdensome in the case of a high-resolution image. In fact, performing background subtraction on compressed images, such as MPEG images, is not novel. In [14], the authors performed background subtraction on a MPEG-compressed video by using the DC-DCT coefficients of image frames. Toreyin *et al.*[15] similarly used this technique on wavelet representation. However, our technique focuses on CS imaging data, not on compressed video files. Moreover for motion tracking algorithms, Kalman filter, particle filter and mean shift methods are often used for tracking motion targets. However higher data dimensionality may be detrimental to the real time performance of tracking, which will lead to greater computational complexity when performing the density and background model estimations.

Compared with the information that is ultimately of use, researchers have begun to consider whether such a large amount of image data is substantially necessary. New motion target detection and tracking strategies need to be developed. With the emergence of CS theory, researchers have begun to engage in motion detection and tracking algorithms by using CS data. For example, [16] describes a method to directly recover background subtracted images by using the CS theory. A single Gaussian distribution background model is employed and a compressive single-pixel camera is used to obtain the compressive sampling images. However the researchers need to recover the original image to update the background model and a single-pixel camera is used to obtain compressive images, which is time consuming and unsuitable for dynamic scenes imaging. In [17], compressive measurements of a surveillance video sequence are decomposed into a low rank matrix and a sparse matrix. The low rank matrix represents the background model, and the sparse components are utilized to identify the moving objects. The augmented Lagrangian alternating direction method is employed to solve the low rank and the sparse matrix simultaneously. However this algorithm requires a video sequence to identify the moving targets, which cannot be used in real time applications. In [18], authors propose a signal tracking algorithm the use compressive observations. The signal being tracked is assumed to be sparse and with slow changes. Compressive measurements are obtained by projecting the known signal *x*_i_ onto a matrix Φ*_i_*, which retains only the columns of Φ with indices that lie in *x*_i_. A Kalman filter in the compressive domain is utilized to estimate signal changes. This algorithm is only suitable for stationary or slowly-moving objects in surveillance scenarios. Wang *et al*. [19] developed a compressive particle filtering algorithm for moving targets tracking with compressive measurements to avoid image reconstruction procedures. Recently, Mei *et al*. [20] proposed a robust *l*_1_ tracker. Each motion target is expressed as a sparse representation of multiple pre-established templates. The *l*_1_ tracker demonstrates promising robustness compared with a number of existing trackers. However computational complexity hinders its real time applications.

## 3. Coded Aperture CI Array

**Figure 1.**
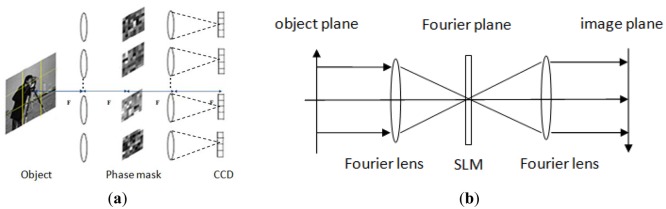
(**a**) Optical compressive imaging system. (**b**) A typical 4F optical system.

Developing practical optical systems to exploit CS theory is a significant challenge. Researchers have proposed several CS imaging architectures and have tested these architectures in the laboratory (see Section 2.2). As Stern proposed in [21], the typical size of a conventional image is megapixels (*N* = 10^6^). For CI system it needs to store the projection matrix Φ*^M^*^×^*^N^*, which is *M* times larger than *N* and can reach 10^12^ maximally. Data storage and the computation for Equation (6) will be challenge. Furthermore to calibrate projection matrix Φ*^M^*^×^*^N^*, *N* point spread functions have to be measured, which is exhaustive and time consuming. In order to solve the aforementioned problems, we propose a coded aperture array optical system to realize CS imaging. Figure 1(a) shows the architecture of our CI system. The general design is based on a 4f system, which comprises of a Fourier transform lens array, an inverse Fourier transform lens array and the corresponding phase-coded masks located between these two lens arrays. For each phase coded 4f system (see Figure 1(b)), the first lens is a Fourier lens, on the focus plane of the Fourier lens it produces a frequency spectrum of the light beam corresponding to the Fourier transformation. Placing a spatial light modulator on this plane to modulate the phase of lights, a phase coded “frequency image” can be obtained. After that we use another Fourier lens to transfer the modulated frequency spectrum to spatial image domain. Thus through a phased coded 4f system, the scene we wish to image can yield a phase coded measurements on detector elements, and finally can be digitally post processed to reconstruct the original scene. For a megapixel image, if we consider a 9 × 9 4f subsystem, the original image will be separated into 9 × 9 blocks. For each block, the image data will be 1/81 of the original image. Therefore the stored sensing matrix Φ_B_*^MB^*^×^*^NB^* (*M_B_* ≪ *N_B_*) of each block will be at least 1/81 × 1/81, which is only 1/6561 of a single aperture CI system. Using separable scheme can effectively reduce the high resolution requirements coded mask needed and facilitate the storage of the coded matrix.

For each 4f subsystem, the action of each phase-coded mask can be considered as implementing a linear projection function across a block of original scene. Each block data collected by a compressive imaging 4f subsystem is represented as:

(7) $$y^{B}=D(h*x^{B})$$

where * denotes convolution, *h* is the phase-coding mask, and D is the random sampling operation of the scene. As shown in [22,23], the convolution of *h* with an image *x* can be represented as the application of the Fourier transform to *x* and *h*. In matrix notation, Equation (7) can be expressed as:

(8) $$y^{B}=D(h*x^{B})=D(F^{-1}C_{h}Fx^{B})$$

where *F* is the two-dimensional Fourier transform matrix and *C_h_* is the diagonal matrix of the *F*(*h*). If the matrix production *F*^−1^*C_h_F* satisfies the RIP, we can accurately recover the original image *x^B^* with high probability when the compressive measurements m ≥ *Ck* log(*n*/*k*). After obtaining all CI signals in each 4f subsystem, the block CS algorithm can be used to reconstruct original signals. Thus by designing such a special optical system, we can acquire compressed imaging measurements.

## 4. Motion Objects Detection Based on CS Images

As previously mentioned, our CI system will segment the CS image into small blocks by using lens arrays. In this section we will demonstrate the method by which to detect CS motion targets directly for each CS imaging block without performing any recovery algorithm. This motion detection algorithm in the CS space is robust and has low computational cost, which will make it suitable for embedded systems.

### 4.1. Background Model

For motion detection algorithms background images are generally assumed to be temporally stationary, whereas moving objects or foreground objects change over time. Suppose that *x_b_* and *x_t_* are real background and test images in the scene and *x_d_* is a difference image or a foreground image. Given that the foreground image is composed by those pixels which only differ from background images. Therefore the foreground image is always smaller than the background image, and can be considered as a sparse signal in a special transformation domain. Suppose that we obtain compressive measurements *y_b_* of training background images *x_b_* and *y_t_* the compressed measurements of current images, the compressive measurements of the foreground image *y_d_* can be expressed as:

(9) $$y_{d}=y_{t}-y_{b}=\Phi x_{t}+n_{t}-(\Phi x_{b}+n_{b})=\Phi x_{d}+n_{d}$$

where *n_t_* is an additional Gaussian noise of *y_t_*, *n_b_* and *n_d_* are the noises of *y_b_* and *y_d_* respectively. By solving a *l*_2_ – *l*_1_ minimization problem [4–5]:

(10) $${\hat{x}}_{d}=\arg\min\frac{1}{2}{\left\|y_{d}-\Phi x_{d}\right\|}_{2}^{2}+\lambda {\left\|\Psi^{T}x_{d}\right\|}_{1}$$

The foreground image *x_d_* can be exactly recovered. In Equation (10), ψ can be the wavelet basis which is always used as the sparse basis. Although detecting moving objects in the compressive domain can be easily achieved by using a background subtraction algorithm and recovering the foreground image in the real world space with *l*_2_ – *l*_1_ minimization, reconstructing the foreground image frame by frame is time consuming. Can we detect the moving object directly in the compressive domain without recovering the foreground image? If the answer is positive, it will dramatically reduce the computational cost and energy consumption of surveillance systems.

The Gaussian background model is often used to segment the foreground and background region in conventional motion detection algorithms. Each pixel (*x*, *y*) over a time series *t* = 1,2……*T* is modeled by a Gaussian distribution *I*(*x*, *y*) ∼ *N*(*u*, *σ*^2^*I*). *σ*^2^*I* is the covariance matrix of the Gaussian model, and *N* is a Gaussian probability density function. According to the Gaussian theorem, if *M*_1_, *M*_2_ are two independent Gaussian random variables, with means *μ*_1_, *μ*_2_ and standard deviations *σ*_1_, *σ*_2_, then their linear combination will also be Gaussian distributed $aM_{1}+bM_{2}\sim N(a\mu_{1}+b\mu_{2},a^{2}\sigma_{1}^{2}+b^{2}\sigma_{2}^{2})$. Therefore it is reasonable to assume each compressive measurement with a Gaussians distribution $N(y_{i},\sigma_{i}^{2}I)$. Here the mean value is *y_i_* = Φ*_i_x*. When the scene changes to include an object that was not part of the background model, theoretically every compressive pixel value *y_i_*, *i* = 1,2……*m* will be against the existing Gaussian distributions. In order to handle image acquisition noise and illumination changes, we use a mixture Gaussian distribution [24,25] to model the background of compressive images and a simple threshold test to declare motion targets.

Using K Gaussian distributions, the probability density function of each compressive measurement at time *t* can be expressed as:

(11) $$P(y_{i,t})=\sum_{j=1}^{k}w_{i,j,t}\times p(y_{i,t},\mu_{i,j,t},\sum_{i,j,t})$$

where *w_i_*_,_*_j_*_,_*_t_*, *μ_i_*_,_*_j_*_,_*_t_* and Σ*_i_*_,_*_j_*_,_*_t_* are the estimates of the weight, mean value, and covariance matrix of the *j* th Gaussian distribution of the *i* th pixel at time *t* in the mixture model respectively. The *j* th Gaussian probability density function *p*(*y_i_*_,_*_t_*, *μ_i_*_,_*_j_*_,_*_t_,* Σ*_i_*_,_*_j_*_,_*_t_*) is defined as:

(12) $$p(y_{i,t},\mu_{i,j,t},\sum_{i,j,t})=\frac{1}{{\left(2\pi \right)}^{\frac{n}{2}}{\left|\sum_{i,j,t}\right|}^{\frac{1}{2}}}\exp(-\frac{1}{2}{\left(y_{i,t}-\mu_{i,j,t}\right)}^{T}\sum_{i,j,t}(y_{i,t}-\mu_{i,j,t}))$$

when a compressive measurement belongs to one Gaussian distribution, its weight parameter *w_i_*_,_*_j_*_,_*_t_* will be large and the standard deviation *σ_i_*_,_*_j_*_,_*_t_* will be small, which indicates that the measurement belongs to a distribution with high certainty. In this paper, the background model parameters *w_i_*_,_*_j_*_,_*_t_*, *μ_i_*_,_*_j_*_,_*_t_* and Σ*_i_*_,_*_j_*_,_*_t_* are estimated by using EM algorithm [26].

### 4.2. Background Model Update

With static background and lighting, only additional Gaussian noise is incurred in the sampling process, the density of background image could be described by a Gaussian distribution centered at the mean pixel value. However most surveillance videos involve lighting changes, shadows, slow moving objects and objects introduced to or removed from the scene. It is very necessary to update the background model continuously. Otherwise, errors in the background accumulate over time and finally trigger unwanted detections.

To update the background, the background parameter of pixel *y_i,t_*_+1_ at time instant *t* + 1 can be estimated by using following equations:

(13) $${\hat{w}}_{i,j,t+1}=(1-\alpha )w_{i,j,t}+\alpha$$

(14) $${\hat{\mu }}_{i,j,t+1}=(1-\rho )\mu_{i,j,t}+\rho y_{i,t+1}$$

(15) $${\hat{\sum }}_{i,j,t+1}=(1-\rho )\sum_{i,j,t}+\rho \Delta \sum_{i,j,t}$$

where *α* is the leaning rate and the parameter *ρ* = *N*(*y_t_*_+1_,*μ_j_*,Σ*_j_*) If the pixel *y_i,t_*_+1_ matches one of the K distributions and is declared as the foreground, then that matched distribution is updated as defined above. Otherwise, the distribution with the smallest weight is discarded, and initialized to this pixel's value.

### 4.3. Motion Detection Based on Compressive Sampling Images

As described in [27], at time *t* the K distributions of the background model are ordered in descending order based on $\frac{w_{j,t}}{\sigma_{j,t}}$. This ordering supposes that a background pixel corresponds to a high weight with a weak variance due to the fact that the background is more static and the background pixel value is practically constant. The first B Gaussian distributions which exceed a certain threshold *T* are considered a background distribution:

(16) $$B=\arg\underset{b}{\min}(\sum_{j=1}^{b}w_{j,t}>T)$$

The other distributions are considered to represent a foreground distribution. At time *t* + 1, if a pixel matches a Gaussian distribution of any B distribution, this pixel will be identified as “background”, otherwise the pixel is classified as “foreground”. If no match is found with any of the K Gaussians, the pixel is also classified as “foreground”. We declare that there is a new object when the result of Equation (17) is above a threshold.

(17) $$E_{y}=\sum_{i=1}^{M}\sum_{j=1}^{k}{\left\|\frac{y_{i}-\mu_{i,j}}{\sigma_{i,j}}\right\|}^{2}$$

## 5. Motion Objects Tracking Based on CS Images

### 5.1. CS-l_1_ Tracking Algorithm

The *l_1_* tracker proposed by the authors in [20] is a promising motion target tracking algorithm, which can handle occlusions, corruption, and lighting changes issues. Their algorithm is based on a particle filter framework and each tracking target *x^T^* ∈ ℝ*^d^* is sparsely represented in a feature dictionary *A* ∈ ℝ*^d^*^×(^*^Nt^*^+2^*^d^*^)^ spanned by target template sets *T* ∈ ℝ*^d^*^×^*^Nt^* and noises templates sets [*I* −*I*] as:

(18) $$x^{T}=[T,I,-I]\left[\begin{array}{c} a\\ e+\\ e- \end{array}\right]=Ac$$

They use particle filter to estimate the posterior distribution $p(s_{t}|x_{t}^{T})$. The state variable *s_t_* is modeled by affine transformation parameters of a target object at time *t*, and the observation *x_t_* is the corresponding object cropped from images by using *s_t_* as parameters. Let *S* = {*s*^1^, *s*^2^, …, *s^n^*} be the *n* state candidates and *X^T^* = {*x^T^*^1^, *x^T^*^2^, …*x^Tn^*} be the corresponding target candidates at time *t*. The target candidate is estimated by finding the smallest projection errors:

(19) $${\hat{x}}^{T}=\arg\max_{x^{T}\in X^{T}}\prod_{j=1,\ldots ,d}\mathbb{N}(x^{T}-Ac)(j);0;\sigma^{2})$$

An *l_1_* optimization algorithm is used to solve the sparse coefficient *c* as follows:

(20) $$\hat{c}=\arg\min\frac{1}{2}{\left\|x^{T}-Ac\right\|}_{2}^{2}+\lambda {\left\|c\right\|}_{1}$$

A template update scheme is subsequently employed to reduce the drift. The main problem of the *l_1_* tracker is the extremely high dimensionality of its feature dictionary space, which leads to a heavy computation burden. Inspired by their outstanding work, we aim to accelerate their tracking algorithm and discuss its application in CI systems. According to Equation (18), in the context of CS the corresponding compressive measurements *y^T^* of *x^T^* can be represented by:

(21) $$y^{T}=\Phi 'x^{T}=\Phi 'Ac$$

where Φ′ ∈ ℝ*^m^*^×^*^d^* is a projection matrix. Obviously, the sparse coefficient *c* in Equation (21) can also be recovered with high probability by using TV optimization algorithm [28], OMP algorithm [29], gradient projection algorithms [30], LARS algorithm [31], and other *l*_1_ – *l*_2_ algorithms:

(22) $$\hat{c}=\arg\min\frac{1}{2}{\left\|y^{T}-\Phi 'Ac\right\|}_{2}^{2}+\lambda {\left\|c\right\|}_{1}=\arg\min\frac{1}{2}{\left\|y^{T}-Dc\right\|}_{2}^{2}+\lambda {\left\|c\right\|}_{1}$$

The feature dictionary *A* in Equation (18) is substituted by a sparse projection dictionary *D* = Φ′ *A*, which can be considered as a compressive measurement of original feature dictionary *A*. As [20] does, the sparse feature dictionary D should also be updated to avoid drift. Clearly, the dimension of dictionary *D* ∈ ℝ*^m^*^×(^*^Nt^*^+2^*^d^*^)^ (*m* ≪ *d*) is reduced by using the random projection matrix Φ′. This will significantly speeds up the process of solving Equation (22).

### 5.2. Compressive Target Image in CI system

**Figure 2.**
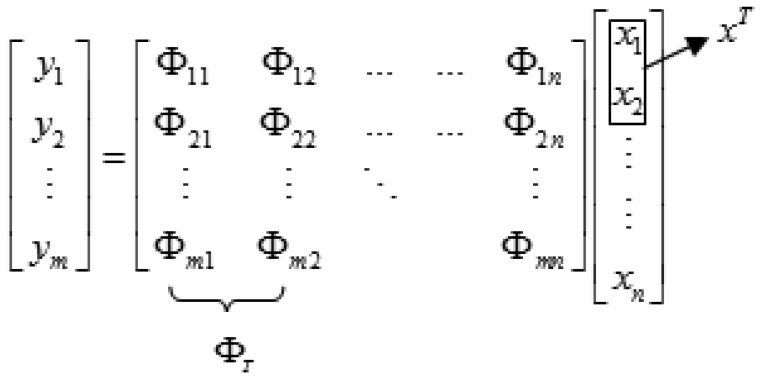
Calculation of CS motion target.

**Figure 3.**
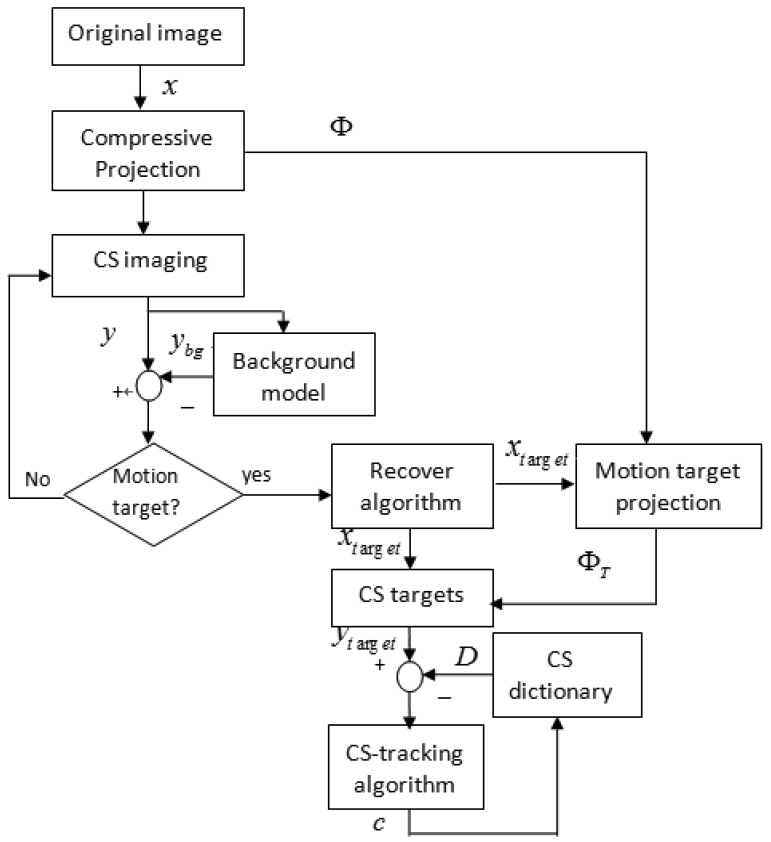
Detection and tracking framework using CS images.

After observing Equation (21), we have a intuitive idea, whether the compressive measurements *y^T^* can be found in a CI system. Suppose that the motion target *x^T^* has been detected through our motion detection algorithm and then reconstructed and labeled (see Figure 2), then we can utilize a projection matrix Φ*_T_* to obtain compressive measurements image *y^T^*. Here Φ*_T_* is a projection matrix by only keeping those columns of Φ whose indices lie in *x^T^*. For our CI system, the projection matrix Φ can be accurately identified by an optical calibration method. Therefore, given the location index of motion targets, the projection matrix Φ*_T_* can be acquired. However, with the movement of target *x^T^*, the projection matrix Φ*_T_* changes as well. In order to simplify our tracking algorithm, the projection matrix Φ′ used in Equation (21) is fixed. The compressive dictionary *D* can be constructed with these compressive target templates. Figure 3 illustrates our motion detection and tracking framework that uses CS sampling images.

## 6. Experiments

### 6.1. Optical System Simulated in Matlab

**Figure 4.**
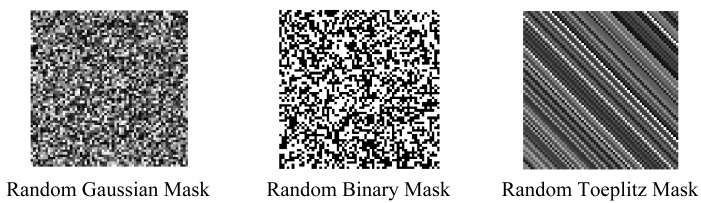
Different mask types.

**Figure 5.**
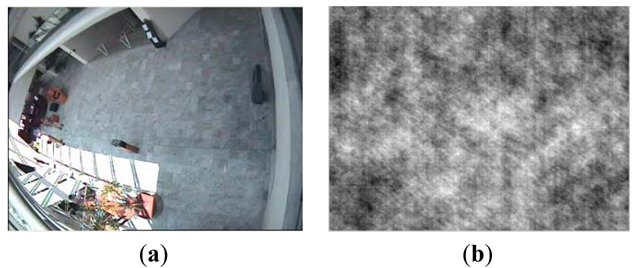
Original image and the corresponding compressive image via Matlab simulation platform. (**a**) Original image; (**b**) CS image using random Gaussian phase coded mask.

Romberg has proven that the random Toeplitz or Gaussian matrix is incoherent with any orthonormal basis ψ with high probability [32]. In [33], a random binary matrix is also proven to be suitable for a projection matrix. Therefore in our experiments, random Gaussian, Toeplitz and binary matrixes are all utilized for phase coded masks. The CAVIAR database provided by INRIA Labs at Grenoble [34] is utilized as original image sequences. In an outdoor sequence, each frame has a size of 288 × 384 with dynamic range [0,255] and motion objects have been generated manually. Figure 4 shows three different phase coded masks we used in our simulation experiments. The corresponding compressive image using random Gaussian phase mask via Matlab simulation is shown in Figure 5.

### 6.2. Performance of Reconstruction Algorithm

**Figure 6.**
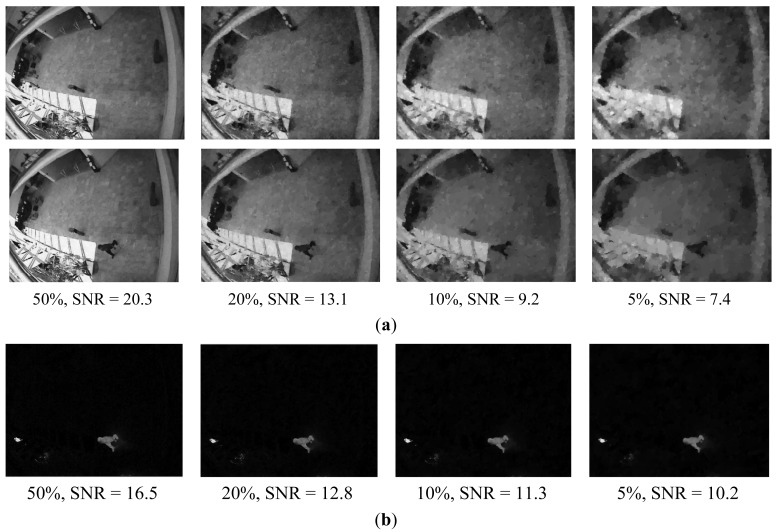
(**a**). Reconstruction of background images and test images with sampling rates from 50% to 5%, and iterations = 800. (**b**). The foreground compressive image reconstructed with sampling rates from 50% to 5% and iterations = 800.

A total variation (TV) optimization algorithm is used to reconstruct the original image from compressive measurements [28]. The reconstruction is performed using several measurement rates ranging from 50% to 5% and with random Gaussian, Toeplitz and binary phase coded masks, respectively. In our experiments, the signal-to-noise ratio (SNR) is applied to evaluate reconstruction performance. Figure 6 shows the reconstruction results with a random Gaussian phase mask.

From Figure 6(a), we can see that the measurement rate can reduce to 20% without sacrificing performance. While a further decreasing measurement rate, the performance is gradually reduced. With rates as low as 5%, the background and test images are not recovered accurately. Figure 6(b) shows the reconstruction results of foreground *y_d_*. We can clearly find in Figure 6(b) that the sparser foreground can be recovered correctly from *y_d_* with rates as low as 5%. These simulation results can be explained by the following assumptions: when the sizes of moving objects are smaller than the original image sizes, we can assume that the sparsity of the motion image *K_d_* is smaller than *K_b_* and *K_t_*. According to the CS theory, the number of compressive measurements necessary to reconstruct original image can be given by *K*log(*N*/*k*). Therefore, if *K_d_* < *K_b_* ≈ *K_t_*, the number of compressive measurements will be smaller than the background and test images.

Table 1 compares the reconstruction results by using different phase coded masks. Here, the sampling rate decreased from 100% to 5%, the same TVAL recovery algorithm is utilized to reconstruct the original image, and the SNR is taken as the average of 10 tests. According to Table 1, the reconstruction algorithm that employs random Gaussian and Toeplitz masks achieves superior recoverey performances than a random binary mask.

### 6.3. Performance of Motion Detection Algorithm

**Figure 7.**
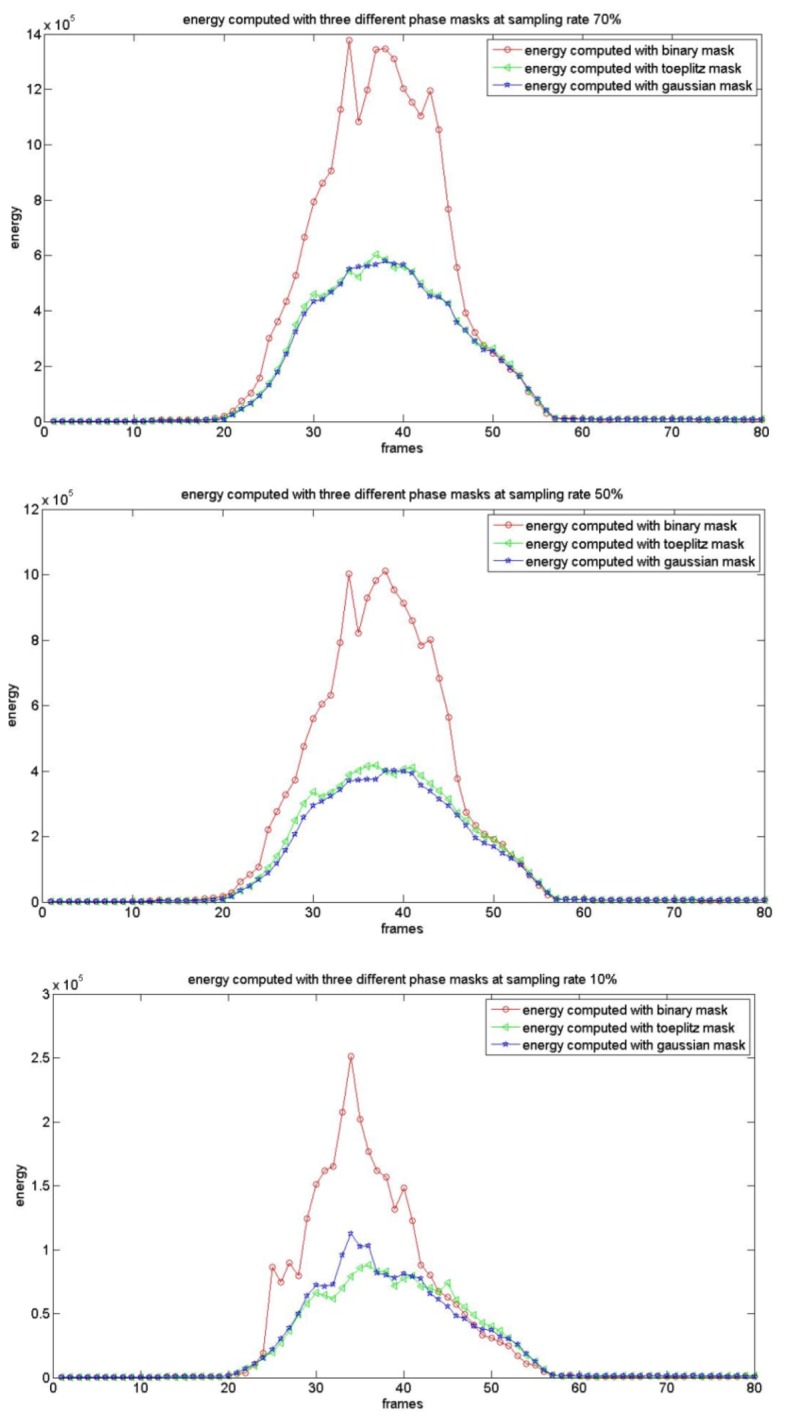
Energy curves computed in a 64 × 64 CI block using different phase masks with sampling rate 70%, 50% and 10% respectively.

As presented earlier, we utilize a mixture Gaussian distribution to model the background. The foreground detection algorithm described in Section 4.3 is used to declare motion objects in compressive sampling space. The motion detection algorithms that use random binary, Gaussian, and Toeplitz phase masks are denoted by RB, RG, and RT respectively in this paper. Figure 7 shows the energy curves computed by using Equation (17) for three different phase mask systems with sampling rates of 10%, 50% and 70% in a 64 × 64 CI block (which included a motion target). Comparing random Gaussian, Toeplitz and binary projections, the energy value collected of compressive measurements is ordered as *E_binary_* > *E_gaussian_* >*E_toeplitz_*. With the decrease of the sampling rate, the energy values computed by using different phase coded masks all reduced gradually. The CS image is declared to include motion targets by using following equation:

(23) $$\begin{array}{c} \text{If}\log E_{y}\geq threshold,\text{motion target}=\text{true}\\ \text{Otherwise}\log E_{y}<threshold,\text{motion target}=\text{false} \end{array}$$

where *thershold* = log(*E_bu_* + *Cσ*), *E_y_* is the energy computed by using Equation (17), and *E_bμ_* is the mean energy of the background CS image. *σ* is the standard variance of *E_μ_* and *C* is a constant.

We employ the Area Under Curve (AUC) metrics to evaluate the performance of our motion detection algorithm. Table 2 shows that the AUC values are affected by the constant *C.* The motion detection performance is the best with constant *C* = 8. Meanwhile the motion detection performance of RB is slightly better than that of RG and RT. The reconstruction performance of RG and RT is better than RB (see Table 1). This observation can be explained by the CS theory. In [32], researchers have proven that random Gaussian and random Toeplitz is incoherent with almost all sparse basis Ψ and thus can recover compressive signals with high possibility. While the binary matrix we used in our experiments are 0–1 matrices, which has been shown that 0,1-matrices require more than O (k log (n/k)) rows to satisfy the RIP [35]. Therefore when the sparsity of the original image is fixed, we need more compressive measurements to recover original signals by using a random binary mask.

### 6.4. Performance of Our Motion Tracking Algorithm

#### 6.4.1. Tracking Efficiency

**Figure 8.**
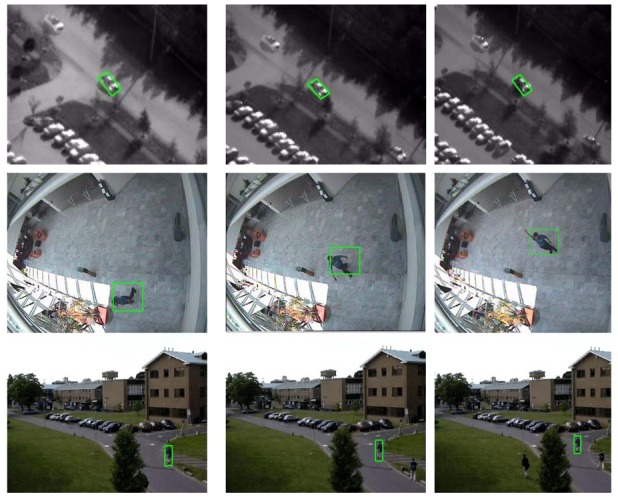
The tracking results with our CS tracker.

To evaluate the performance of our tracking algorithm, three videos were used in the experiments. The first test sequence is an infrared (IR) image sequence that was also used in [20]. CAVIAR [34] and PET2001 databases [36] were also used to examine our algorithm in terms of efficiency and accuracy. In our experiments, a random Gaussian projection matrix was performed with the dictionary dimension reduced from 100% to 83%, 55%, 22% and 10%. We retained the other experimental parameters as in [20]. In Table 3 we recorded the elapsed time of the *l*_1_ tracker and our CS tracker for each test experiment. According to Table 3, our CS tracker is 4–5 times faster than *l*_1_ tracker, even without dimensional reduction operation. With the decrease in sampling rates, our CS tracker is 10 times faster than *l*_1_ tracker. Figure 8 shows our tracking results with three video sequences.

From the experimental results we can seen that the computation of our CS-*l*_1_ tracking algorithm is much cheaper. First, the reduction of templates' dimensionality would speed up the optimization process. Second, probably the most important reason is that our method can lower the rank of feature dictionary matrix *A*. Mathematically, *rank*(*AB*) ≤ min {*rank*(*A*), *rank*(*B*)}, therefore *rank*(*D* = Φ*A*) ≤ *rank*(*A*). The rank of our CS-*l*_1_ tracker is smaller than that of *l*_1_ tracker, which accelerates the rate of iteration convergence obviously and hence makes it faster than its counterpart.

#### 6.4.2. Tracking Accuracy

**Figure 9.**
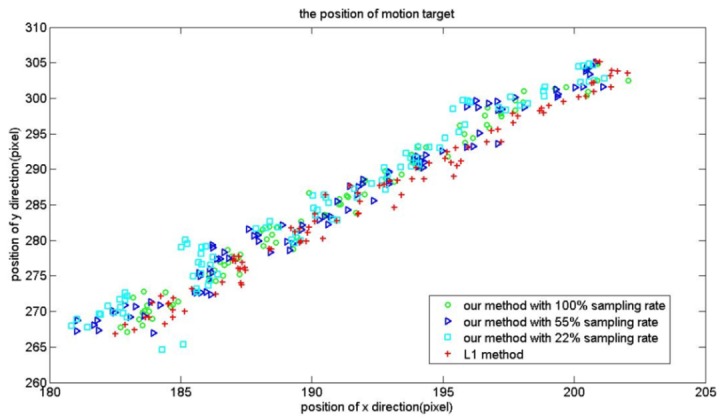
The position of motion targets computed by using our method and *l*_1_ tracker for pets sequences.

Intuitively, with the reduction of the sampling rate the tracking accuracy will decrease. Thus we also examine the tracking accuracy of our tracker with *l*_1_ tracker. For the PetsD2 video sequence, the red points are the trajectories of the motion target computed by using the *l*_1_ tracker. Cyan, blue and green points are positions computed using our method with a sampling rate from 22%, 55% to 100%. As illustrated in Figure 9, the tracking approaches achieve similar performance on the video sequence with a sampling rate of 100%. With the decrease in sampling rates, the position error gradually increased.

## 7. Conclusions

We have demonstrated that by using a CI system we can detect and track objects in motion with significantly fewer data samples than conventional image methods. A parallel coded aperture imaging array, which is based on a phase-coded 4F system, is used to simulate compressive sensing images. A Gaussian mixture model is generated off-line for later use in on-line foreground detection directly in the compressive domain and a TV optimization algorithm is used for image reconstruction. A real-time CS tracking algorithm is proposed and then applied using compressive sensing images. For compressive imaging system, experimental results show that with the decrease in measurement rates, the recovered image performance is gradually reduced. Compared with the random binary mask, simulation results show that the use of random Gaussian or Toeplitz phase masks can achieve high resolution reconstructed images. Motion detection experimental results demonstrate that low dimensional compressed imaging representation is sufficient to determine spatial motion targets. The minimum amount of measurements to perform motion detection algorithm in compressive domain is fewer than the number of measurements needed to recover background and the test image. Motion tracking results show that we can construct a compressive dictionary and use it as a template set in the CS image space. With the same *l*_1_ reconstruction algorithm, our CS tracking method is 10 times faster than *l*_1_ tracking method.

## Figures and Tables

**Table 1.** Reconstruction performance with different phased coded mask styles.

**SNR**	**100%**	**70%**	**50%**	**30%**	**10%**	**5%**
Binary	32	15.9	13	10.3	7.2	5.7
Gaussian	32.1	26.6	20.3	14.4	9.2	7.4
Toeplitz	32	25.7	19.5	14.1	9.0	7.3

**Table 2.** AUC for motion detection using different thresholds and 50%, 10% sampling rates.

**AUC**	**RB (50%)**	**RG (50%)**	**RT (50%)**	**RB (10%)**	**RG (10%)**	**RT (10%)**
*th* = log(*E_bu_* + 6*σ*)	0.975	0.8875	0.9375	0.9625	0.825	0.8
*th* = log(*E_bu_* + 8*σ*)	0.975	0.9625	0.9625	0.95	0.9625	0.9625
*th* = log(*E_bu_* + 15*σ*)	0.9375	0.95	0.95	0.925	0.95	0.95

**Table 3.** The running speed of *l*_1_ tracker and our CS tracker with 300 particles.

	**L1 tracker**	**Our 100%**	**Our 83%**	**Our 55%**	**Our 22%**	**Our 10%**
IR image	4.6 s	1 s	0.77 s	0.56 s	0.50 s	0.45 s
CAVIAR	4.79 s	0.91 s	0.68 s	0.61 s	0.55 s	0.51 s
Pets	5.14 s	0.72 s	0.63 s	0.57 s	0.51 s	0.47 s
